# Three-Dimensional Arterial Spin Labeling-Guided Sub-Volume Segmentation of Radiotherapy in Adult Non-Enhancing Low-Grade Gliomas

**DOI:** 10.3389/fonc.2022.914507

**Published:** 2022-07-01

**Authors:** Zihong Zhu, Guanzhong Gong, Lizhen Wang, Ya Su, Jie Lu, Yong Yin

**Affiliations:** ^1^ Department of Oncology, Affiliated Hospital of Southwest Medical University, Luzhou, China; ^2^ Department of Radiation Oncology Physics and Technology, Shandong Cancer Hospital and Institute, Shandong First Medical University and Shandong Academy of Medical Sciences, Jinan, China

**Keywords:** low-grade gliomas, non-enhancing, sub-volume segmentation, three-dimensional arterial spin labeling, radiomics

## Abstract

**Objective:**

The present study aimed to evaluate the feasibility of sub-volume segmentation for radiotherapy planning of adult non-enhancing low-grade gliomas (NE-LGGs) guided by three-dimensional arterial spin labeling (3D-ASL). The differences in high- and low-perfusion areas of NE-LGGs were analyzed using multi-sequence magnetic resonance imaging (MRI) radiomics.

**Methods:**

Fifteen adult patients with NE-LGGs were included in the study. MR images, including T1-weighted imaging (T1WI), T2 Propeller, T2 fluid-attenuated inversion recovery (T2 Flair), 3D-ASL, and contrast-enhanced T1WI (CE-T1WI), were obtained. The gross tumor volume (GTV) was delineated according to the hyperintensity on T2 Flair. The GTV was divided into high- and low-perfusion areas, namely GTV-ASL and GTV-SUB, respectively, based on the differences in cerebral blood flow (CBF) value. The volumes and CBF values of high- and low-perfusion areas were measured and compared. The least absolute shrinkage and selection operator (LASSO) regression was used to select the optimal features of all MR maps. Receiver operating characteristic (ROC) curve analysis was used to evaluate the diagnostic accuracy of the absolute CBF_mean_ (aCBF_mean_), relative CBF_mean_ (rCBF_mean_, normalized by the CBF value of the normal gray matter), and screened features in differentiating high- and low-perfusion areas.

**Results:**

Among the enrolled patients, three (20%) patients with NE-LGGs showed focal intra- and post-radiotherapy contrast enhancement within a prior high-perfusion area of 3D-ASL. The volume ratio of the GTV-ASL to the GTV was (37.08% ± 17.88)% (46.26 ± 44.51 vs. 167.46 ± 209.64 cm^3^, *P = *0.000). The CBF_mean_ in the high-perfusion area was approximately two times of that in the edema area or normal gray matter (66.98 ± 18.03 vs. 35.19 ± 7.75 or 33.92 ± 8.48 ml/100g/min, *P = *0.000). Thirteen features were screened, seven of which were extracted from 3D-ASL. The area undercurve (AUC) values of aCBF_mean_, rCBF_mean_, and firstorder_10Percentile from 3D-ASL were more than 0.9, of which firstorder_10Percentile was the highest. Their cut-off values were 44.16 ml/100 g/min, 1.49 and 31, respectively.

**Conclusion:**

The difference in blood perfusion in the GTV can be quantified and analyzed based on 3D-ASL images for NE-LGGs, which could guide the sub-volume segmentation of the GTV. 3D-ASL should become a routine method for NE-LGGs during simulation and radiotherapy.

## 1 Introduction

Low-grade gliomas account for approximately 15% of all gliomas, which are the most common primary brain tumors in adults (1, 2). Non-enhancing LGGs (NE-LGGs) are a special type of LGGs. These NE-LGGs were not enhanced on conventional enhanced computed tomography (CT)/magnetic resonance imaging (MRI). Their blood vessel wall composition is similar to that of brain-healthy tissue, with a complete blood-brain barrier. Macromolecules used for tumor enhancement cannot pass through the blood-brain barrier to enhance these lesions. Therefore, the lesions show low density on enhanced CT and T1 hypointensity and T2 hyperintensity on enhanced MRI [the manifestation of edema (3, 4)]. Eichberg et al. (5) showed that approximately 20% of gliomas were not enhanced, of which more than 70% were LGGs. LGGs enhanced as commonly as they lacked enhancement (6).

Radiotherapy is an effective treatment for NE-LGGs. At present, the abnormal hyperintensity (edema area) on T2 fluid-attenuated inversion recovery (T2 Flair) is usually used as the standard for delineating the tumor target volume, which often makes the gross tumor volume (GTV) too large. The tumor boundary of NE-LGGs is difficult to identify in conventional imaging, and the blood flow and metabolism of the tumor are significantly different from those of the brain tissue in the edema area in biology (7). Quantifying this difference through functional imaging can clarify the distribution of tumor cell enrichment areas, which could guide the delineation of target volume during radiotherapy. Commonly functional imaging requires intravenous administration of a contrast agent and is therefore somewhat limited.

One method of obtaining perfusion contrast without the need for an external contrast agent is three-dimensional arterial spin labeling (3D-ASL), which generates an image by magnetically labeling water protons of arterial blood as an endogenous tracer. It is not influenced by the destruction of the blood–brain barrier and can reflect microscopic changes such as tissue blood perfusion and micro-vessel density (8, 9). ASL is attractive as not only is a contrast agent application not required, but cerebral blood flow (CBF) can also be quantified in absolute values [ml/min/100 g brain tissue (10)]. Some studies have shown that CBF obtained by ASL perfusion imaging has a significant positive correlation with micro-vessel density (*ρ* = 0.567) and the expression of vascular endothelial growth factor (*r* = 0.604) and a significant negative correlation with survival (*r* = -0.714). Multivariate regression analysis showed that CBF was an independent risk factor for overall survival [OS; HR = 1.028, 95% CI: 1.005–1.051, *P = *0.010 (11, 12)]. ASL has been used to guide stereotactic biopsy and operation for glioma. Jin et al. (13) showed that compared with conventional MRI, combined magnetic resonance spectroscopy (MRS) and ASL improved the accuracy of target selection for the stereotactic biopsy of intracranial tumors, especially in three cases, each of low-enhancing and non-enhancing gliomas. Lindner et al. (14) concluded that intraoperative arterial spin-labeling was a feasible, reproducible, and reliable tool to map CBF in brain tumors and seemed to give beneficial information compared to conventional intraoperative MRI in partial resection.

The quality of ASL-derived perfusion maps has reached a level that makes the method useful for many clinical and research applications. A consensus white paper regarding recommended implementation of arterial spin-labeled perfusion MRI for clinical applications has been established, in which pseudo-continuous labeling and a segmented 3D readout were recommended (15). 3D-ASL perfusion imaging can effectively differentiate intracranial tumors from non-neoplastic lesions (16). It has been widely used in diagnosing, grading, and the efficacy evaluation of gliomas (17, 18). However, few reports are on its application in guiding the delineation of radiotherapy targets for NE-LGGs. Thus, we segmented the sub-volume based on the perfusion difference of the GTV in 3D-ASL for radiotherapy planning of NE-LGGs. Radiomics is an emerging technique that can provide more detailed quantification in multi-sequence MR studies. It is also defined as the conversion of images to higher-dimensional data and the subsequent mining of that data. We also analyzed the characteristic differences of high- and low-perfusion areas of NE-LGGs using multi-sequence MRI radiomics and obtained the steady characteristics that could distinguish high- and low-perfusion areas of NE-LGGs. It provides a reference for the clinical definition of radiotherapy target for patients with NE-LGGs and lays a theoretical basis for precision radiotherapy.

## 2 Materials and Methods

### 2.1 Patients

This retrospective study was approved by the Institutional Review Board of Shandong Cancer Hospital and informed consent forms were obtained from all patients prior to enrollment. Fifteen adult patients with pathologically confirmed glioma from Shandong Cancer Hospital were analyzed between December 2018 and May 2021. Patients showed nonenhancement on diagnostic brain MRI that strongly suggested a diagnosis of World Health Organization (WHO) grade I or II glioma. Radiological studies performed on the patients were accessed by two Neuroradiologists from Picture Archiving and Communication Systems (PACS). WHO grading was reviewed by three neuropathologists according to the fifth edition of the WHO Classification of Tumors of the Central Nervous System [WHO CNS5 (19)]. Patient characteristics are presented in **Table 1**. All enrolled patients received radical radiotherapy with 45–60 Gy in 1.8–2.0 Gy fractions. All but three patients received concurrent temozolomide chemotherapy.

**Table 1:** Clinical characteristics of all patients.

Characteristics	Number of patients (%)
Age (mean ± SD, year; range)	49 ± 14 (31–74)
Gender	
Female	5 (33%)
Male	10 (67%)
Tumor location	
Unilateral	7 (47%)
Midline^a^ (H3 K27-altered status)	5 (33%)
Altered	0 (0%)
Non-altered	5 (33%)
Bilateral (widely diffuse distribution)	3 (20%)
Type of histopathology	
Astrocytoma	15 (100%)
WHO classification	
Grade 2	15 (100%)
IDH mutation status	
Mutant	1 (7%)
Wild type	10 (67%)
NOS	4 (26%)
Median follow-up time^b^, Median (range)	12 months (1–29 months)

^a^Midline location includes brainstem, thalamus, cerebellum. ^b^The follow-up method was done according to RANO criteria (20). SD, standard deviation. NOS, not otherwise specified. IDH, isocitrate dehydrogenase.

### 2.2 MRI Protocol

#### 2.2.1 Before Radiotherapy

MRI was performed on a 3.0-T superconducting MR scanner (Discovery 750W, GE Healthcare, USA) equipped with an 8-channel head coil. The head of a patient was fixed with a thermoplastic film. A series of MRI sequences, including T1-weighted imaging (T1WI), T2 Propeller, T2 Flair, 3D-ASL, and contrast-enhanced T1WI (CE-T1WI), were performed for each patient with a slice thickness of 3 mm and no interslice gap width. The common parameters of these sequences are shown in **Table 2**. For CE-T1WI, gadopentetate dimeglumine was power-injected at doses standardized according to the patient’s body weight (0.2 ml/kg) at 2 ml/s, and the scan was started at 3–5 min after injection.

**Table 2:** The common parameters of MRI sequences.

Parameters	T1WI	T2 Propeller	T2 FLAIR	CE-T1WI	3D-ASL
TR (ms)	8.5	13,500	11,000	8.5	5,160
TE (ms)	3.2	114	120	3.2	11.5
FOV (cm)	25.6 × 25.6	26 × 26	26 × 26	25.6 × 25.6	25.6 × 25.6
Matrix	256 × 256	416 × 416	320 × 320	256 × 256	512 × 512
Flip angle (°)	12	120	160	12	110

T1WI, T1-weighted imaging; T2 FLAIR, T2 fluid-attenuated inversion recovery; 3D-ASL, three-dimensional arterial spin labeling; CE-T1WI, contrast-enhanced T1WI; TR, repetition time; TE, echo time; FOV, field of view.

#### 2.2.2 3D-ASL Data Acquisition and Post-Processing

Set the labeling plane on the neck in advance and use pseudo-continuous labeling, image acquisition with single-shot gradient-echoecho planar imaging (EPI), labeling duration = 1,500 ms, post-labeling delay (PLD) time = 2,025 ms, the number of excitation (NEX) = 3. The scan time was approximately 4.5–5 min. The parameter values of repetition time (TR), echo time (TE), field of view (FOV), matrix, and flip angle are shown in **Table 2**. Control/label and calibration images were acquired. For measurement of the magnetization of arterial blood and also for segmentation purposes, an M0 calibration image was acquired separately with the same geometry and the same imaging parameters as the ASL without labeling. Background suppression technology was used in the process of image acquisition to reduce physiological noise and motion artifacts. The quantitative CBF maps were generated automatically using GE FuncTool 4.7 software after scanning based on the following equation (21):


$$\text{CBF }=\frac{6000\cdot \text{λ}\cdot \left({\text{SI}}_{\text{control}}-{\text{SI}}_{\text{label}}\right)\cdot {\text{e}}^{\frac{\text{PLD}}{{\text{T}}_{1,\text{blood}}}}}{2\cdot \text{α}\cdot {\text{T}}_{1,\text{blood}}\cdot {\text{SI}}_{\text{PD}}\cdot \left(1-{\text{e}}^{-\frac{\text{τ}}{{\text{T}}_{1,\text{blood}}}}\right)}\left[\text{ml}/100\text{ g}/\text{min}\right]$$

Where λ is the brain/blood partition coefficient in ml/g, SI_control_ and SI_label_ are the time-averaged signal intensities in the control and label images respectively, T_1,blood_ is the longitudinal relaxation time of blood in seconds, α is the labeling efficiency, SI_PD_ is the signal intensity of a proton density-weighted image, τ is the label duration, and PLD is the post-labeling delay. The following parameters were used in this study: λ = 0.9, PLD = 2,025 ms, T_1,blood_ = 1,650 ms, α = 0.85, and τ = 1,500 ms. The SI_PD_ was calculated using echo planar imaging M0 images. A factor of 6,000 converts the units from ml/g/s to ml/100 g/min, which is standard in the physiological literature.

#### 2.2.3 During Radiotherapy

At the 10th–15th radiotherapy, the patient again underwent MR scanning. MRI sequences, machine, and parameters were the same as before radiotherapy.

### 2.3 The Definition and Delineation of the Target Volume

Two senior radiologists used MIM Maestro software (version 6.8.8, USA) to delineate the target volume, which was then reviewed by a third senior radiologist. Axial T2 Flair sequences were used to contour the hyperintense signal abnormality as the GTV. The GTV-ASL was defined as a high-perfusion area under the guidance of a CBF map derived from 3D-ASL sequence that was free of cysts, necrosis, calcifications, hemorrhage, and large vessels. The GTV-SUB was the low-perfusion area of GTV, which was obtained by subtracting the GTV-ASL from the GTV. In addition, the boundary of target volume was also determined in conjunction with other conventional sequences such as T1WI, T2 Propeller, and CE-T1WI. Finally, the GTV-ASL and GTV-SUB were copied to the corresponding sequences (T1WI, T2 Propeller, T2 Flair, 3D-ASL, and CE-T1WI) to prepare for later feature extraction.

### 2.4 CBF Values Measurement

T2 Flair and 3D-ASL images were fused using rigid registration in a workstation with commercially available software (GE Healthcare, ADW 4.7, USA). According to the pseudo-color perfusion of 3D-ASL images, the regions of interest (ROIs) were set at the slice with the highest perfusion signal while avoiding areas of cysts, necrosis, calcifications, hemorrhage, and large vessels. The area of high perfusion was defined as ROI-T. Four ROIs were selected in the edema area around the high-perfusion area and defined as ROI-E. The ROI of brain tissue (ROI-N) was selected from the contralateral mirror gray matter of the lesion (the unilateral cerebral hemisphere) or normal gray matter of the leftinsulalobe (midline or widely diffuse distribution). The ROIs area of the peritumoral edema area and normal gray matter were 100–150 mm^2^. The absolute CBF values (aCBF), including maximum CBF values (CBF_max_), minimum CBF values (CBF_min_) and average CBF values (CBF_mean_) of the high-perfusion areas and CBF_mean_ of the edema areas and normal gray matter, were measured, respectively. The CBF values of the high-perfusion area and edema areanormalized by that of normal gray matter (namely relative CBF values, rCBF) were also calculated to correct for age-dependent and patient-dependent variations of cerebral perfusion.

### 2.5 MR Images Data Pre-Processing

The pre-processing procedure was undertaken before features extraction, including co-registration, denoising, field bias correction, and resampling. All maps were firstly co-registered to T1WI anatomic images. Then, T1WI images were normalized to the Montreal Neurological Institute (MNI) space, which generated a transformed matrix from the native space to the MNI space. Each image was then spatially smoothed with a 6-mm full-width at a half-maximum Gaussian kernel to decrease spatial noise. In order to compensate for non-uniform intensity caused by field inhomogeneity, N4ITK MRI bias field correction was applied to each imaging study. Isotropic resampling to a voxel size of 1 × 1×1 mm^3^ was performed. All pre-processing was done using MATLAB 2020b software (MathWorks Inc., Natick, MA, USA, http://www.mathworks.com/products/matlab/).

### 2.6 Features Extraction

All images were transmitted into 3D slicer software (version 4.8.1, http://www.slicer.org,USA). The radiomics features of the GTV-ASL and GTV-SUB mapping areas from five different MR sequences (T1WI, T2 Propeller, T2 Flair, 3D-ASL, and CE-T1WI) were extracted, including morphological features (*n* = 14), first-order features (*n* = 18), gray level co-occurrence matrix (GLCM, *n* = 24), gray level dependence matrix (GLDM, *n* = 14), gray level run length matrix (GLRLM, *n* = 16), gray level size zone matrix (GLSZM, *n* = 16), and neighborhood gray-tone difference matrix (NGTDM, *n* = 5). A total of 535 features were obtained.

### 2.7 Features Selection

Feature selection was conducted using python (version 3.8.8, https://www.python.org). Before the features selection process, all the features were normalized to the range of [0, 1] for standardization so that features of different orders of magnitude could be reasonably compared. The variance threshold method was performed to pre-process the features, which first calculated the variance of each feature and then removed features with a variance lower than 0.8 (22). The least absolute shrinkage and selection operator (LASSO) regression was used to select the optimal features. In order to avoid over-fitting, the parameters of the LASSO regression were tuned to select the key features from the high-dimensional feature space using five-fold cross-validation.

The LASSO regression is a shrinkage method that can actively select from a large and potentially multicollinear set of variables in the regression, resulting in a more relevant and interpretable set of predictors (23). LASSO performs *via* a continuous shrinking operation, minimizing regression coefficients in order to reduce the likelihood of overfitting; however, the technique is computed so as to shrink the sum of the absolute value of regression coefficients, forcing and producing coefficients that are exactly 0; thus, selecting for the nonzero variables to remain. The complexity of LASSO is controlled by λ. The larger λ is, the greater the penalty will be for the linear model with more variables and, finally, a model with fewer variables.

### 2.8 Statistical Analysis

**Figure 1:**
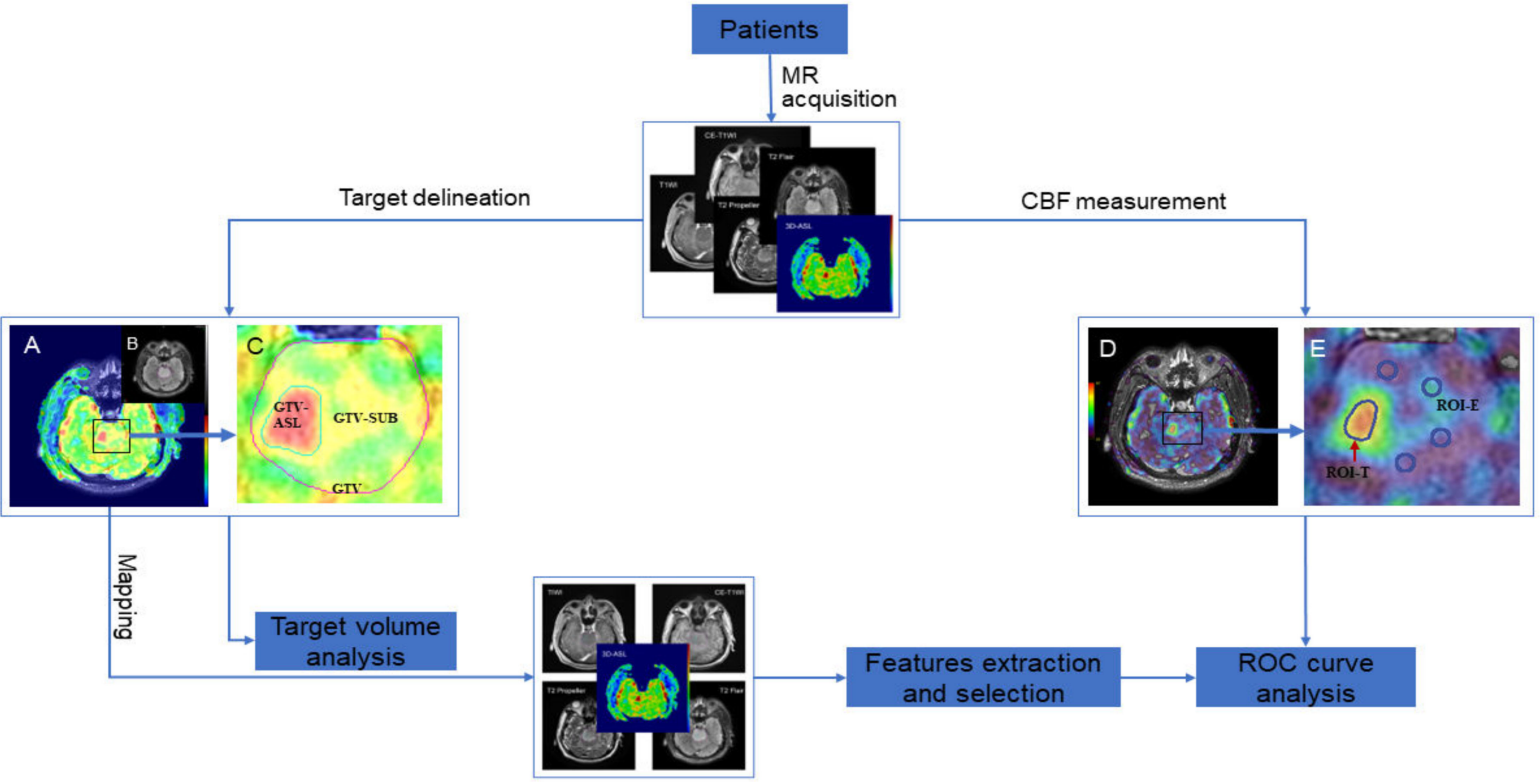
Flow chart of this study. **(A, D)**, fusion images of T2 Flair and 3D-ASL; **(B)** T2 Flair image; **(C, E)**, local magnification of **(A, D)**, respectively.

SPSS (version 25.0, IBM, NY, USA) was used for statistical analysis. Paired t-test or Wilcoxon test was used to compare the target volumes and the CBF values, depending on the distribution defined by the Shapiro–Wilk normality test. Receiver operating characteristic (ROC) curve analysis was performed by using MedCalc Statistical Software version 20.010 (MedCalc Software Ltd, Ostend, Belgium; https://www.medcalc.org; 2021), which evaluated the diagnostic accuracy of the aCBF_mean_, rCBF_mean_ and screened features in distinguishing high- and low-perfusion areas. Data was presented as mean ± standard deviation. All statistical tests were two-tailed, and a *P* value of <0.05 was considered to be significant. The flow chart of this study is shown in **Figure 1**.

## 3 Results

### 3.1 The Comparison of Target Volumes

The volumes of the GTV and GTV-ASL were 167.46 ± 209.64 cm^3^ and 46.26 ± 44.51 cm^3^, respectively. The volume ratio of the GTV-ASL to the GTV was (37.08% ± 17.88)% (*Z *= –3.408, *P *= 0.001, Wilcoxon test). The high-perfusion area of 3D-ASL did not exceed the abnormal hyperintense area (edema area) of T2 Flair for all patients.

### 3.2 CBF Values

#### 3.2.1 Before Radiotherapy

The aCBF and rCBF values of sub-volumes before radiotherapy in all patients are shown in **Table 3**. The aCBF_mean_ in the high-perfusion area was approximately two times of that in the edema area (*t = *7.934, *P = *0.000) or normal gray matter (*t = *8.368, *P = *0.000), but the CBF_mean_ values were comparable between the edema area and normal gray matter (*t = *0.495, *P = *0.625).

**Table 3:** The CBF values of sub-volume before radiotherapy (ml/100 g/min).

	High-perfusion area			Edema area	Normal gray matter
	CBF_max_	CBF_min_	CBF_mean_	CBF_mean_	CBF_mean_
Absolute values	89.25 ± 39.50	42.33 ± 11.79	66.98 ± 18.03	35.19 ± 7.75	33.92 ± 8.48
Relative values	2.61 ± 0.64	1.34 ± 0.53	2.03 ± 0.53	1.08 ± 0.32	–

CBF_max_/CBF_min_/CBF_mean_ represent the maximum, minimum, and average value of cerebral blood flow (CBF), respectively. Relative values are the CBF values of high-perfusion and edema area normalized by that of normal gray matter.

#### 3.2.2 Comparison of CBF Values Before and During Radiotherapy

Six out of 18 patients underwent MR scanning again at the 15th–20th session of radiotherapy. Compared with those before radiotherapy, the CBF_max_, CBF_min_, and CBF_mean_ of the high-perfusion area and the CBF_mean_ of the edema area increased by (12.47 ± 46.35)%, (6.55 ± 28.52)%, (11.46 ± 22.13)%, and (6.44 ± 29.65)%, respectively, and the CBF_mean_ in normal gray matter decreased by (5.07 ± 11.76)%. But there was no significant difference in all changes (*P* *>* 0.05). **Table 4** shows the details of CBF values.

**Table 4:** CBF values before and during radiotherapy (ml/100 g/min).

	High-perfusion area			Edema area	Normal gray matter
	CBF_max_	CBF_min_	CBF_mean_	CBF_mean_	CBF_mean_
Before radiotherapy	77.17 ± 17.45	45.83 ± 9.47	66.09 ± 13.45	38.05 ± 7.81	40.44 ± 12.28
During radiotherapy	86.00 ± 35.79	50.67 ± 11.64	69.31 ± 16.96	40.23 ± 12.57	38.14 ± 12.60
Test statistic	-0.647	-1.336*	-0.314	-0.492	1.077
*P* values	0.546	0.239	0.753	0.644	0.331

CBF_max_/CBF_min_/CBF_mean_ represent the maximum, minimum, and average values of cerebral blood flow (CBF), respectively. The asterisk (*) represents the test statistic of Wilcoxon test and the others are t-test values.

### 3.3 Features Selection

**Figure 2:**
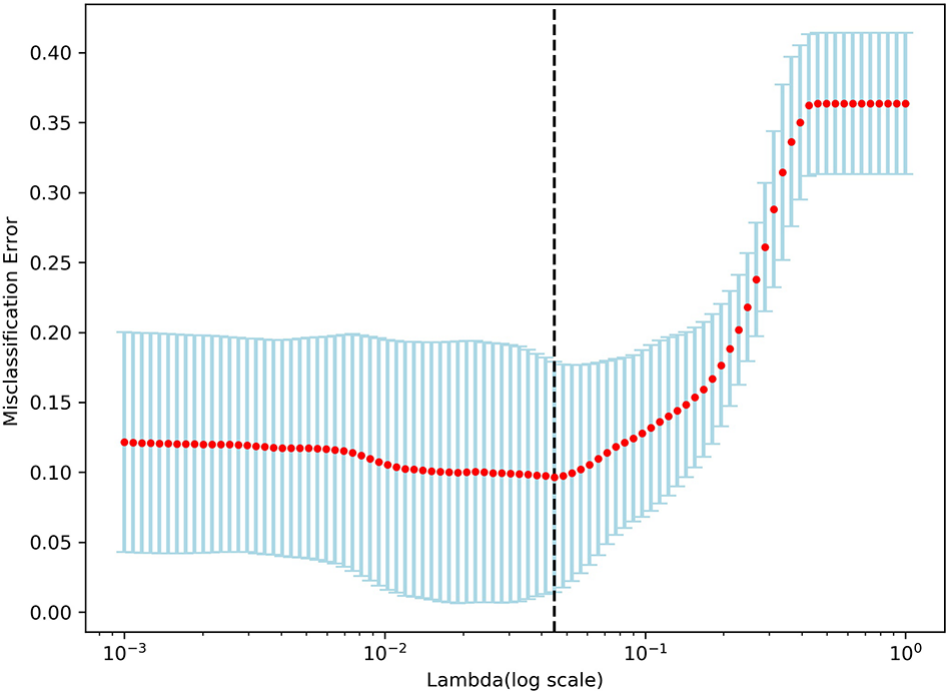
Tuning parameter (λ) selection in the LASSO regression.

**Figure 3:**
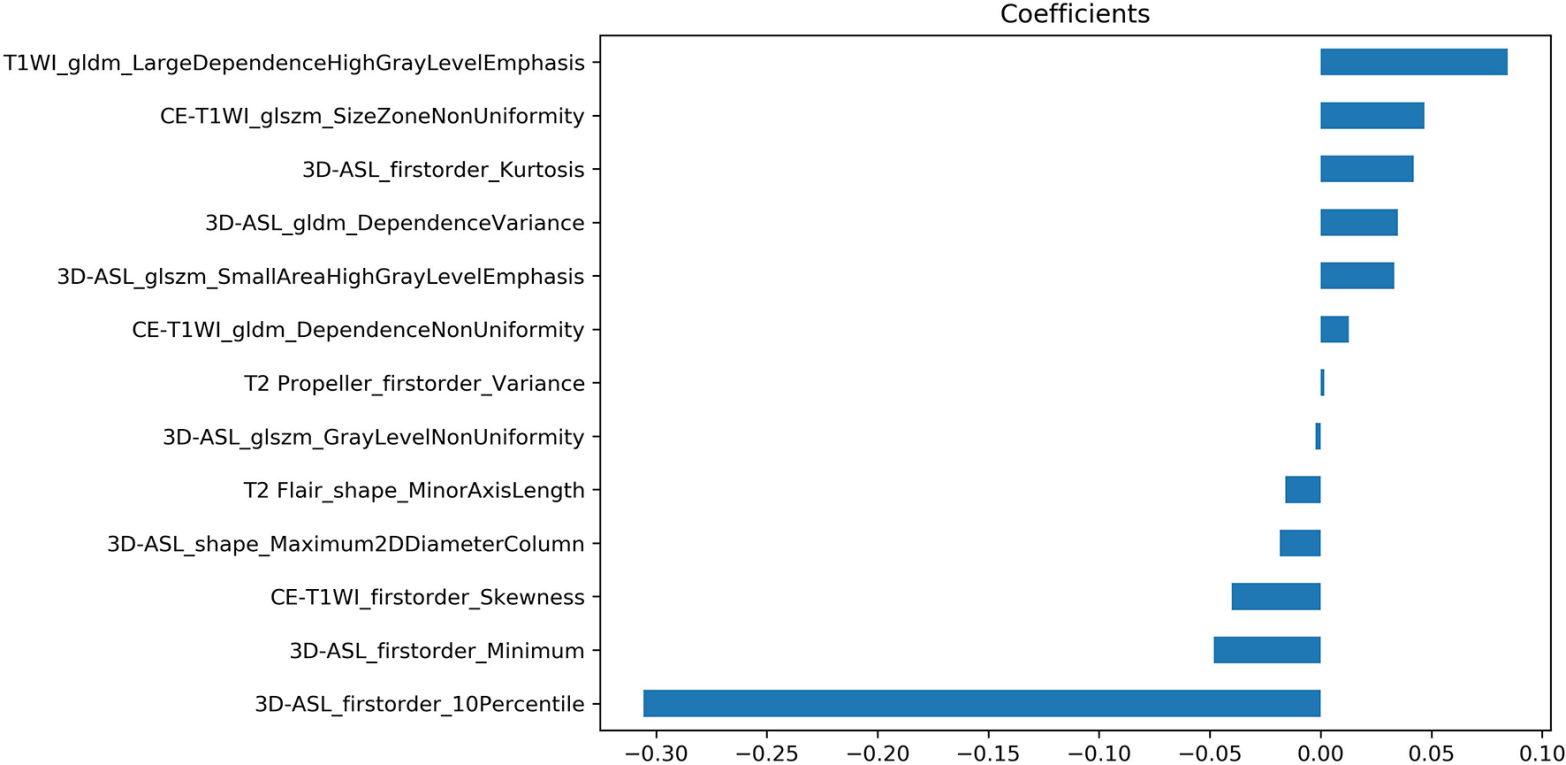
The coefficients of screened features using the LASSO regression.

A total of 298 features were retained after screening by the variance threshold method, including 63 features in T1WI, 61 features in CE-T1WI, 56 features in 3D-ASL and 59 features in each of T2 Propeller and T2 Flair, respectively. Based on the LASSO regression, 13 steady features were screened out of the 298 features to differentiate high-and low-perfusion areas. The minimum criteria for five-fold cross-validation were applied during λ selection. The misclassification error was generated versus λ, as shown in **Figure 2**. As λ was equal to 0.04484, which was defined as the optimal λ value with a minimal misclassification error, only 13 features, potentially the most contributing to differentiating high-and low-perfusion areas, remained in the model. Further, 7 out of 13 features were derived from 3D-ASL, as shown in **Figure 3**.

### 3.4 ROC Curve Analysis

**Figure 4:**
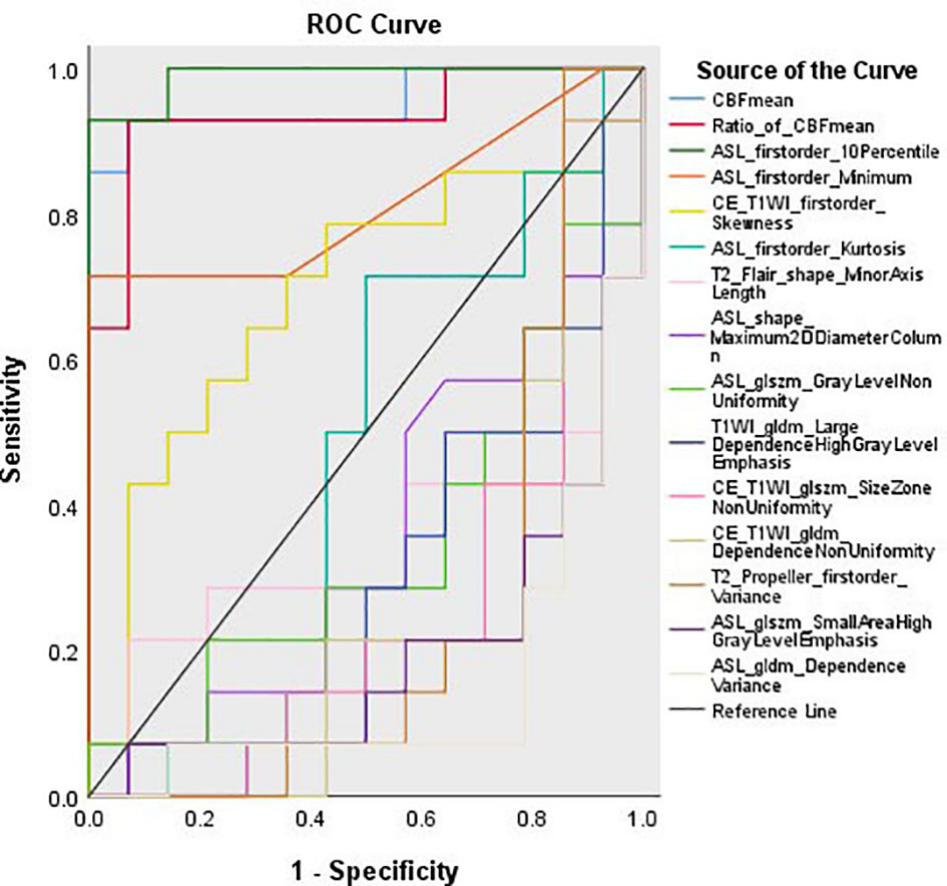
Receiver operating characteristic (ROC) curves.

The area under curve (AUC) values of the aCBF_mean_ and rCBF_mean_ were 0.954 and 0.934, respectively. The cut-off values of aCBF_mean_ and rCBF_mean_ were 44.16 ml/100 g/min and 1.49, which yielded 92.86% sensitivity and 92.86% specificity. The AUC value of firstorder_10Percentile from 3D-ASL was 0.990, which was the highest among the 13 remaining features and higher than that of the aCBF_mean_ and rCBF_mean_. The feature with a cut-off of 31 yielded 92.86% sensitivity and 100% specificity. The ROC curve is shown in **Figure 4**.

### 3.5 Spatial Distribution Between Post-Treatment Focal Contrast Enhancement and Prior High-Perfusion Area

**Figure 5:**
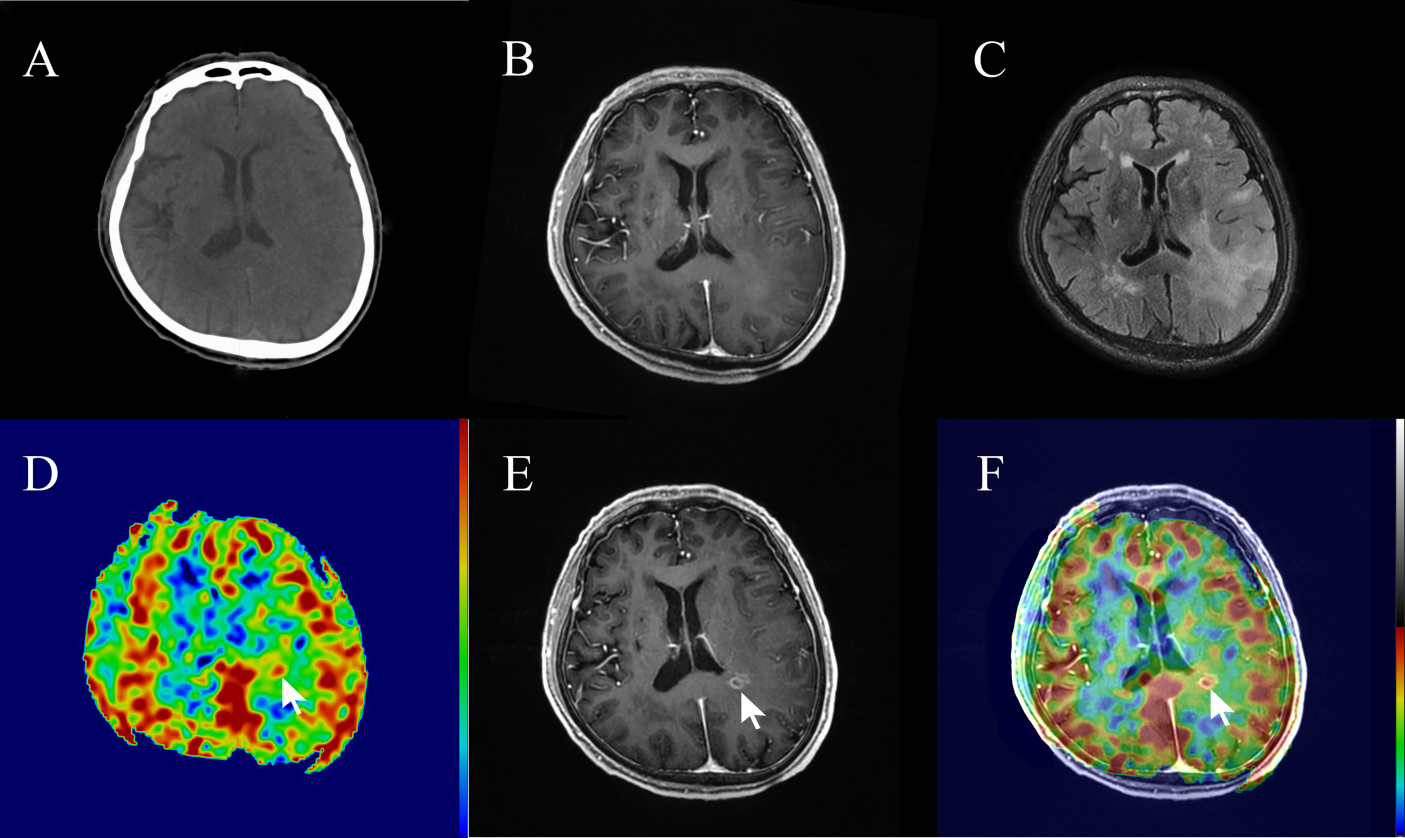
A 74-year-old patient with non-enhancing low-grade glioma (WHO grade II astrocytoma) showed focal contrast enhancement within a prior non-enhanced and high-perfusion area at 20 sessions of radiotherapy (40 Gy). **(A)** Planning plain-CT image. **(B)** Contrast-enhanced T1-weighted imaging (CE-T1WI) before radiotherapy. **(C)** T2 fluid-attenuated inversion recovery (T2 Flair) image before radiotherapy. **(D)** Three*-*dimensional arterial spin labeling (3D-ASL) image before radiotherapy. **(E)** CE-T1WI at 20 sessions of radiotherapy. **(F)** Fusion image of **(D, E)**.

**Figure 6:**
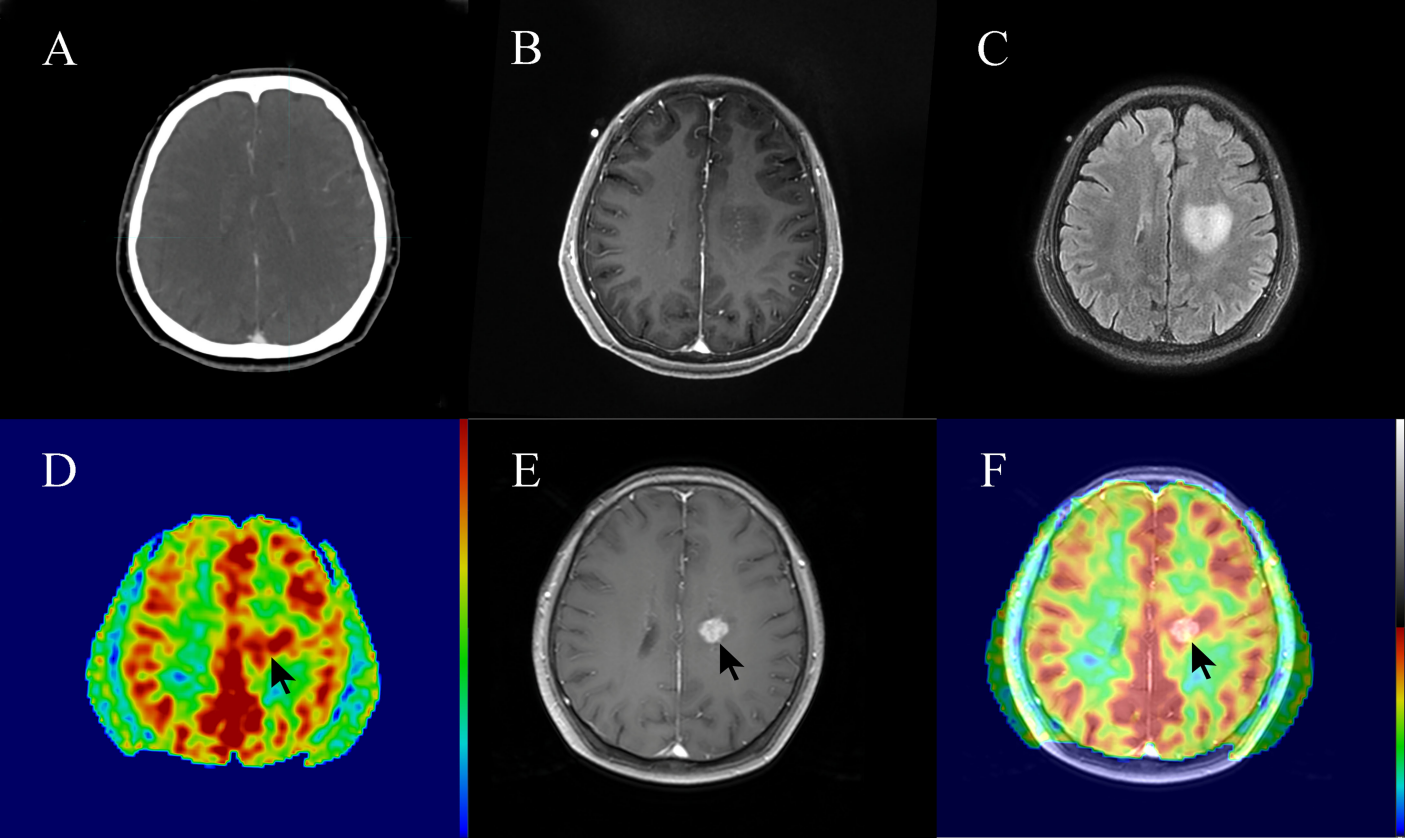
A 62-year-old patient with non-enhancing low-grade glioma (WHO grade II astrocytoma) showed focal post-radiotherapy contrast enhancement within a prior non-enhanced and high-perfusion area at 3 months following radiotherapy. The patient had clinical symptoms such as weakness of the right lower limb, considering the tumor progression. **(A)** Planning contrast-enhanced CT (CE-CT) image. **(B)** Contrast-enhanced T1-weighted imaging (CE-T1WI) before radiotherapy. **(C)** T2 fluid-attenuated inversion recovery (T2 Flair) image before radiotherapy. **(D)** three-dimensional arterial spin labeling (3D-ASL) image before radiotherapy. **(E)** CE-T1WI at 3 months following radiotherapy. **(F)** Fusion image of **(D, E)**.

In this study, 3 out of 15 patients (20%) with high-perfusion in 3D-ASL prior to radiotherapy showed new gadolinium contrast enhancement on MRI assessment during radiotherapy or at 3 months post-radiotherapy (**Figures 5**, **6**).

## 4 Discussion

Most LGGs inevitably develop into high-grade malignant gliomas, and even with effective treatment, the survival rate of patients with LGGs remains poor (24, 25). Tom et al. (26) showed that the incidence of malignant transformation of LGGs was 17%, and the median OS time after malignant transformation was only 2.4 years. As a special type of LGGs, NE-LGGs show no enhancement on CT and MR. If the edema area is used as the main standard for GTV delineation, there will be great blindness. The research of Jakola et al. (27) showed that the blood–brain barrier was destroyed after the malignant transformation of NE-LGGs, and the enhanced area was much smaller than the edema area. Moreover, Tatekawa et al. showed that Flair hyperintense volume for IDH wild-type gliomas showed no significant association with OS (28). If the edema area is defined as the GTV of NE-LGGs, which can ensure the radiation dose coverage of tumor, the radiation damage risk to the surrounding healthy tissues will be increased due to the large.

Dynamic susceptibility contrast (DSC)-MRI, CT perfusion imaging, single-photon emission tomography (SPECT), and H2[15O] positron-emission tomography (PET) measure perfusion by dynamic imaging of the passage of a contrast agent. The physical basis of ASL offers its greatest advantage over traditional contrast bolus techniques: it is non-invasive. ASL does not require a gadolinium-based tracer, eliminating the risk of nephrogenic systemic fibrosis in patients with renal dysfunction (29). ASL is also favorable for pediatric populations as it avoids the technical difficulties and ethical problems of contrast agents and radiation exposure with CT and nuclear medicine techniques (30). In addition, ASL MRI has been extensively validated against other methods that use exogenous contrast agents, such as 15O-PET, and ASL implementations are now commercially available on all major MRI platforms, with demonstrated reproducibility in multi-center studies (31–34). Thus, we conducted a sub-volume segmentation for the GTV of NE-LGGs based on the CBF perfusion difference derived from 3D-ASL.

Our study found that the lesions of all patients showed no enhancement on CE-T1WI, while hyperintensity on T2 Flair and high local perfusion on 3D-ASL were observed. The local high-perfusion area was within the edema area. The analyses of target volumes revealed that the volume of the GTV-ASL (high-perfusion area) was significantly smaller than the GTV. Wyss et al. (35) studied the spatial heterogeneity between the uptake of ^18^F-fluoroethyl tyrosine (18F-FET) and CBF. The results showed that the volumes of increased CBF and ^18^F-FET uptake spatially coincided and were correlated (*rho* = 0.944), and the increase in CBF was more confined to the tumor center. This was similar to the spatial relationship between abnormal hyperintensity on T2 Flair (edema area) and high local perfusion on 3D-ASL for NE-LGGs in this study. Hayes et al. (36) studied the effect of ^18^F-FET PET on the radiotherapy plan of patients with suspected unenhanced glioblastoma. The results showed that the biological tumor volume of ^18^F-FET was greater than the GTV of conventional MRI, which may reduce the risk of tumor omission. The high cost of amino acids for PET limits its wide application in clinical settings, and exogenous tracers are used in PET, which may increase the risk of allergy and renal injury. However, 3D-ASL can make up for these deficiencies.

Local control failure is the main reason for the treatment failure of LGGs. In traditional radiotherapy, most LGGs relapse at the site of the primary tumor after radiotherapy (37, 38). This may be related to insufficient radiation dose or excessive dose to the surrounding healthy tissues due to a very large target area, which limits the radiation dose of the central tumor. Most of the tumors in this study were located in the midline (thalamus, brainstem, or cerebellum) or bilateral (widely diffuse distribution), accounting for approximately 53%, but the volume of the local high-perfusion area was significantly smaller than that of the edema area. The reduction of the target volume can avoid the damage of high-dose radiotherapy to the surrounding healthy tissues, especially for patients whose tumors are located in important functional areas such as the midline or whose lesions are diffusely distributed, which will provide a basis for us to further improve the targeted dose for the sub-volume of high perfusion.

In the present study, the CBF_mean_ values were comparable between the peritumoral edema area and normal gray matter. 3D-ASL can differentiate between invasive and non-invasive peritumoral edema; hence, the infiltration area of tumor for NE-LGGs shows high local perfusion, while the edema area around the high-perfusion area is the non-invasive area of angiogenic edema or gliosis, and its CBF value is equivalent to that of surrounding healthy tissue (39). The present study found that the CBF_mean_ of the GTV-ASL was approximately twice that of the GTV-SUB or normal gray matter. Khashbat et al. (40) measured the tumor blood flow (TBF) of 6 non-enhancing low-grade astrocytomas. The results showed that the ratio of TBFmean to CBF_mean_ of brain-healthy tissue was 0.88 ± 0.41, but the region of interest was delineated based on T2WI, and the TBF was measured after mapping to ASL. In fact, the TBF included the CBF of the tumor and the edema area. Our study, however, divided the GTV into high- and low-perfusion areas based on the perfusion difference in 3D-ASL, and measured the CBF values of sub-volume. Furthermore, the number of cases we enrolled was significantly more than that in their study.

This study compared the changes in CBF before and after radiotherapy. The results showed that compared with that before radiotherapy, the CBF values of tumor and edema area increased, while that of normal gray matter decreased after radiotherapy. Calmon et al. (41) studied the changes in CBF values for 43 children with diffuse intrinsic pontine gliomas before radiotherapy, after radiotherapy, treatment progress, and at the last follow-up. The results showed that the ASL-CBF value of all patients after radiotherapy was significantly higher than that before radiotherapy (*P *< 0.001). Petr et al. (42) showed that the perfusion of brain-healthy tissue decreased by -9.8% ± 20.9% (*P *= 0.032) after radiotherapy. Our results are consistent with the trend of CBF noted in these studies.

It is difficult for conventional MRI to identify the nonenhanced tumor or the micro-invasive lesion around the enhanced tumor, but the microscopic differences between the tumor and the peritumoral edema area can be found by imaging radiomics. Yan et al. (43) reported that multimodal MRI radiomics could show different characteristics in the potential progression area of preoperative MRI of glioblastoma. We found that there were significant features in the high- and low-perfusion areas of NE-LGGs after analyzing the features of multi-sequence MRI. The ROC curve analysis was also used in this study, which found that the diagnostic accuracy of the aCBF_mean_, rCBF_mean_ and firstorder_10Percentile from 3D-ASL in differentiating the high- and low-perfusion areas were more than 0.9, of which firstorder_10Percentile was the highest. Therefore, the combination of radiomics features may be helpful in distinguishing the non-enhanced tumors and peritumoral edema of NE-LGGs. And the optimal cut-off values of aCBF_mean_ and rCBF_mean_weredetermined as 44.16 ml/100 g/min and 1.49, respectively, which lay the foundation for further research on the threshold determination of sub-volume based on 3D-ASL in the future.

In this study, one patient showed no enhancement in CE-T1WI before radiotherapy, but the new enhancement appeared after 20 sessions of radiotherapy (the irradiation dose was 40 Gy). Cao et al. (44) showed that in the non-enhanced tumor region, contrast uptake increased significantly after the receipt of approximately 10 Gy of irradiation (*P* < 0.01), and reached the maximum after the receipt of approximately 30 Gy of irradiation, while the healthy brain showed only nonsignificant changes during and after irradiation. The blood-tumor barrier was more sensitive to radiation; this finding was similar to the results of the present study. Moreover, three patients with NE-LGGs showed the new gadolinium contrast enhancement after radiotherapy in this study, which was consistent with the high-perfusion area of 3D-ASL before radiotherapy, especially in one patient who showed tumor progression. This finding suggests that 3D-ASL is helpful in identifying the infiltration areas of non-enhanced tumors for NE-LGGs.

Our study has some limitations. CBF was initially measured using the general kinetic model, an adaptation of the ASL model described by BUXTON et al. (21). The imperfection of the quantification model and the uncertainty and potential errors of 3D-ASL measured CBF due to the prolonged transit delay between the tagging region and the imaging slice, influenced sub-volume segmentation. Furthermore, GTV and GTV-ASL segmentation was linked to inter-and intra-observer experience and condition.Finally, the sample size was not large enough, and the data had a large degree of dispersion. The next step is to increase the number of cases and apply 3D-ASL to radiotherapy planning to further evaluate the value of this technique in radiotherapy.

## 5 Conclusion

The difference in blood perfusion in the GTV can be quantified and analyzed based on 3D-ASL images for NE-LGGs, which guided the sub-volume segmentation of the GTV. The differences between high-and low-perfusion areas in GTV can be identified by multi-sequence MR radiomics features. 3D-ASL should be used as a routine method for NE-LGGs during simulation and radiotherapy, especially if the contrast agent cannot be injected or when contrast enhancement is uncertain.

## Data Availability Statement

The original contributions presented in the study are included in the article/supplementary material. Further inquiries can be directed to the corresponding author.

## Ethics Statement

The studies involving human participants were reviewed and approved by Institutional Review Board of Shandong Cancer Hospital. The patients/participants provided their written informed consent to participate in this study.

## Author Contributions

GG conceived and designed the study. LW and YS collected data. ZZ performed the experiments, made a statistical analysis, and wrote the paper. YY and JL reviewed and edited the manuscript. All authors contributed to the article and approved the submitted version.

## Funding

This work was supported by the Start-up fund of Shandong Cancer Hospital (YYPY2020-016), and the Key Research and Development Program of Shandong Major Science & Technology Innovation Project (2021SFGC0501).

## Conflict of Interest

All authors declare that the research was conducted in the absence of any commercial or financial relationships that could be construed as a potential conflict of interest.

## Publisher’s Note

All claims expressed in this article are solely those of the authors and do not necessarily represent those of their affiliated organizations, or those of the publisher, the editors and the reviewers. Any product that may be evaluated in this article, or claim that may be made by its manufacturer, is not guaranteed or endorsed by the publisher.

## Abbreviations

LGGs, low-grade gliomas; NE-LGGs, non-enhancing low-grade gliomas; CT, computed tomography; MRI, magnetic resonance imaging; T2 Flair, T2 fluid-attenuated inversion recovery; GTV, gross tumor volume; 3D-ASL, threedimensional arterial spin labeling; CBF, cerebral blood flow; OS, overall survival; MRS, magnetic resonance spectroscopy; PACS, Picture Archiving and Communication Systems; WHO CNS5, the fifth edition of the WHO Classification of Tumors of the Central Nervous System; IDH, isocitrate dehydrogenase; NOS, not otherwise specified; T1WI, T1-weighted imaging; CET1WI, contrast-enhanced T1WI; PLD, post labeling delay; NEX, number of excitation; TR, repetition time; TE: echo time; FOV, field of view; GTV-ASL, the high-perfusion area based on 3D-ASL; GTV-SUB, the low-perfusion area based on 3D-ASL; ROIs, the regions of interest; aCBF, absolute CBF value; rCBF, relative CBF value; MNI, Montreal Neurological Institute; GLCM, gray level cooccurrence matrix; GLDM, gray level dependence matrix; GLRLM, gray level run length matrix; GLSZM, gray level size zone matrix; NGTDM, neighborhood gray tone difference matrix;LASSO, the least absolute shrinkage and selection operator; ROC, receiver operating characteristic; AUC: area under curve;DSC, dynamic susceptibility contrast; SPECT, single-photon emission tomography; PET, positron-emission tomography; 18F-FET, 18F-fluoroethyl tyrosine; TBF, tumorblood flow.
